# Multiple imputation for non-response when estimating HIV prevalence using survey data

**DOI:** 10.1186/s12889-015-2390-1

**Published:** 2015-10-16

**Authors:** Amos Chinomona, Henry Mwambi

**Affiliations:** Department of Statistics, Rhodes University, Grahamstown, South Africa; School of Mathematics, Statistics and Computer Science, University of Kwa-Zulu Natal, Pietermaritzburg, South Africa

**Keywords:** Complete case analysis, Multiple imputation, Missing at random, Design-consistent estimates

## Abstract

**Background:**

Missing data are a common feature in many areas of research especially those involving survey data in biological, health and social sciences research. Most of the analyses of the survey data are done taking a complete-case approach, that is taking a list-wise deletion of all cases with missing values assuming that missing values are missing completely at random (MCAR). Methods that are based on substituting the missing values with single values such as the last value carried forward, the mean and regression predictions (single imputations) are also used. These methods often result in potential bias in estimates, in loss of statistical information and in loss of distributional relationships between variables. In addition, the strong MCAR assumption is not tenable in most practical instances.

**Methods:**

Since missing data are a major problem in HIV research, the current research seeks to illustrate and highlight the strength of multiple imputation procedure, as a method of handling missing data, which comes from its ability to draw multiple values for the missing observations from plausible predictive distributions for them. This is particularly important in HIV research in sub-Saharan Africa where accurate collection of (complete) data is still a challenge. Furthermore the multiple imputation accounts for the uncertainty introduced by the very process of imputing values for the missing observations. In particular national and subgroup estimates of HIV prevalence in Zimbabwe were computed using multiply imputed data sets from the 2010–11 Zimbabwe Demographic and Health Surveys (2010–11 ZDHS) data. A survey logistic regression model for HIV prevalence and demographic and socio-economic variables was used as the substantive analysis model. The results for both the complete-case analysis and the multiple imputation analysis are presented and discussed.

**Results:**

Across different subgroups of the population, the crude estimates of HIV prevalence are generally not identical but their variations are consistent between the two approaches (complete-case analysis and multiple imputation analysis). The estimates of standard errors under the multiple imputation are predominantly smaller, hence leading to narrower confidence intervals, than under the complete case analysis. Under the logistic regression adjusted odds ratios vary greatly between the two approaches. The model based confidence intervals for the adjusted odds ratios are wider under the multiple imputation which is indicative of the inclusion of a combined measure of the within and between imputation variability.

**Conclusions:**

There is considerable variation between estimates obtained between the two approaches. The use of multiple imputations allows the uncertainty brought about by the imputation process to be measured. This consequently yields more reliable estimates of the parameters of interest and reduce the chances of declaring significant effects unnecessarily (type I error). In addition, the utilization of the powerful and flexible statistical computing packages in **R** enhances the computations.

## Background

Most practical survey data, especially those obtained for scientific and social investigations are often characterized by missing data as a result of non-response. In particular non-response is regarded as a pervasive and persistent problem in most social research studies. Most analyses of incomplete data often take a complete-case analysis approach despite the fact that current statistical software resources have capabilities for an enhanced analysis. That is, a list-wise deletion approach in which cases with missing values are omitted from the analysis is adopted by many researchers. This is mainly based on the assumption that missing data are missing completely at random (MCAR) as described by [1]. However this assumption is generally difficult to justify in practice. Furthermore, ad hoc methods that substitute the missing values by plausible values such as the last value carried forward, the mean and regression predictions (single imputation) are also often used. However these methods have considerable drawbacks especially if the percentage of missing data is high as explained by [1, 2]. Biased results can be obtained if the complete data are not representative of the entire sample (MCAR assumption is violated) and also relationships amongst variables are lost. In addition, single imputation may yield unduly small standard errors since the uncertainty about the imputed values is not accounted for [2].

There are several reasons why data are missing in surveys, see for example [1–5]. Missing data may be a result of an element in the target population not being included on the survey's sampling frame, resulting in what is called non-coverage. These elements have zero probability of being selected into the sample as explained by [1, 6, 7]. If a sampled element does not participate in the survey, this results in total/unit non-response. Total non-response may occur because of a participant's refusal to take part in the survey or due to language barrier or non-availability on the day of interview. The success of data collection in surveys, particularly in household surveys relies on the availability of participants on the day of interview. However participants are often unavailable resulting in missing data. Furthermore, a responding sampled element can fail to provide acceptable responses to one or more of the survey items resulting in what is termed item non-response. The reasons for item non-response range from a respondent refusing to answer a question because it is too sensitive or does not know the answer or gives an answer that is inconsistent with answers to other questions [1, 6, 8]. A non-response that falls between unit and item non-response is called partial non-response. Partial non-response occurs when a substantial number of item non-response occurs. This can occur, for instance, when a respondent cuts off the phone call in the middle of an interview or when a respondent in a multiphase survey provides data for some but not all phases of data collection [1, 3, 6].

Missing data are classified according to the relationship between measured variables and the probability of missing data in what [1, 4, 5] termed “missing data mechanisms”. The missing data mechanisms define the distribution of missing data given the underlying data. The missing data can fall into one of three missing data mechanisms namely missing completely at random (MCAR), missing at random (MAR) and missing not at random (MNAR).

Various methods have been developed in an attempt to compensate for non-response in survey data. The form of compensation depends on the source of the missing data. As described by [1, 3, 4] deletion, weighting adjustments and imputation methods are the most common ways used for handling and/or compensating for non-response. In particular, compensation for total non-response and non-coverage is made by weighting adjustments. The respondents are assigned greater weight in the analysis so as to account for the shortfall resulting from the non-respondents. In the case of non-coverage, since the sample provides no information about the missing elements, weighting adjustments are based on external data sources. For the case of item non-response, compensation is done via imputation, see [1]. The imputation method involves systematically filling the missing value with new assigned values. Partial non-response can be compensated by both weighting adjustments and imputation.

Most statistical methods for data analysis assume a rectangular matrix with rows representing units and the columns representing variables measured for each unit. However this is often not the case in most practical scientific and social research including human immunodeficiency virus (HIV) studies due to missing data. The current study illustrates and highlights the multiple imputation technique for handling missing data and obtains unbiased estimates of HIV prevalence in Zimbabwe using socio-economic and demographic variables.

Originally suggested by [1], the multiple imputation method is a Monte Carlo (or simulated based) technique that replaces each missing value with two or more plausible values utilizing a Bayesian inference paradigm. Essentially each missing value is imputed *m* (≥2) different times using the same imputation method creating *m* data sets with no missing values. Each completed data set is analyzed using standard complete-data procedures as if the imputed data were real data obtained from the non-respondents and obtain desired parameter estimates and their respective standard errors. The results are later combined to produce estimates and confidence intervals that incorporate missing-data uncertainty. The combined estimates, called multiple imputation estimates, are obtained by finding the mean of the parameter estimates and variance estimates that account for both the within-imputation and across-imputation variability see [1, 8–13]. The overarching idea is to use the observed values to provide indirect evidence about the likely values of the unobserved ones averaging over the distribution of the missing data given the observed data [2]. Thus for this reason multiple imputation falls under the MAR missingness mechanism as opposed to the MCAR. Key to this lies in correctly specifying the imputation model. In addition, the multiple imputation procedure is a computational intensive analytic approach that accounts for the variability due to the missing values.

Since the multiple imputation method relies on a Bayesian paradigm, a prior distribution for the parameters is required. By default, most software packages utilize the non-informative prior distribution that correspond to a state of prior ignorance about model parameters, [14, 15]. The Bayesian approach employs the Markov chain Monte Carlo (MCMC) procedure to simulate draws from the posterior distribution of the missing data given the observed data, see [1, 14, 15]. The application of the multiple imputation method comes with potential problems that are worthy noting as pointed out by [2]. These include, challenges pertaining to ways for handling non-normally distributed variables, plausibility of the MAR assumption and how to handle data that are MNAR. For the current research, these are adequately accounted for in the statistical package **mi**, as explained in Subsection 2.5 below, that we used for the multiple imputation computations. The research also followed the guidelines outlined in strengthening the reporting of observational studies in epidemiology (STROBE) as outlined in [16]. The MNAR approaches which rely on sensitivity analysis are not the focus of the current application.

The article is organized in the following format. Section 2 gives an overview of the data used for analysis, the underlying concepts of the multiple imputation method, a brief description of the missing data and the statistical computing package used for the analysis. Section 3 presents the results of the analyses in the form of descriptive and logistic regression analyses from both a complete case analysis and a multiple imputation analysis. Section 4 gives a detailed discussion of the findings and strengths and limitations of the research. Section 6 gives the concluding remarks. The aims of the current study is to illustrate and highlight the strength of the multiple imputation as a method of handling missing data and a technique for accounting for the uncertainty about the missing data.

## Methods

### The data

The data used for the study were obtained from the 2010–11 Zimbabwe Demographic and Health Surveys (2010-11ZDHS). The DHSs in general are country-level population-based household surveys. The data from DHS are mainly aimed at providing information for monitoring and impact evaluation of key indicators pertaining to population, health and nutrition. Household data regarding socio-economic, health and demographic variables are collected using questionnaire-based interviews. Specifically, for the 2010-11ZDHS females aged 15 to 49 and males aged 15 to 54 were eligible for interview and collection of blood samples or specimens, using dried blood spot (DBS), for laboratory testing (which includes HIV testing). The data were obtained from the DHS Data Archives, [17].

For HIV testing, blood samples were collected on a special filter paper card using capillary blood from a finger prick. An “anonymized” antibody testing process was conducted at the National Microbiology Reference Laboratory (NMRL) in Harare. Bar coded labels were used to identify the DBS samples to ensure the anonymity and these were used to track the outcome of the testing procedure and the results. Laboratory testing of the blood specimens followed a standard laboratory algorithm designed to maximize the sensitivity and specificity of the test results. In particular, the algorithm uses two different HIV antibody enzyme-linked immunosorbent assays (ELISAs) that are based on antigens. Discordant samples that were positive in the first test were retested using both ELISAs and discordant samples from the second round of testing were regarded as “indeterminate”. The”indeterminate” were then subjected to a western blot confirmatory test, in which the results were considered final. Written consent was sought from the respondents before the collection of the blood samples, and for the 15–17 year old respondents further consent was also sought from their parents or responsible adult. Furthermore, consent was also sought to store blood samples for future research. All participants were given information brochures pertaining to HIV/AIDS and giving details of the nearest facility providing voluntary counseling and testing (VCT). All HIV testing procedures were reviewed and approved by the ethical review boards of ORC Macro, a US-based company that provides technical assistance to DHS worldwide, the Centers for Disease Control (CDC) and the Medical Research Council of Zimbabwe (MRCZ).

Under the 2010-11ZDHS, a stratified two-stage cluster sampling design was used to collect the data using the 2002 population census figures as the sampling frame. Individuals were clustered within households which in turn were clustered within enumeration areas (EAs) and the country's ten administrative provinces were regarded as the strata. For the current research the response variable is HIV status, a binary variable indicating whether a respondent is HIV positive or negative. The socio-economic and the demographic variables (that were used as the predictors) are selected as those factors thought to influence HIV infection. These factors include age, gender, marital status, education level, economic status (household wealth), religion, province and place of residence (whether rural or urban). The sample consists of 17,434 respondents, 14,491 with non-missing value and an additional 2943 with missing values in at least one of the measured variables. Table 1 gives the variables and their respective percentages of missing values.

**Table 1:** Frequencies and percentages of missing values per variable

Variable	Frequency of missing values	% of missing values
HIV Status	2 772	15.90
Gender	0	0.00
Employment Status	220	1.26
Marital Status	762	4.37
Contraception	1 083	6.21
Wealth Index	737	4.23
Literacy Level	174	1.00
Religion	711	4.08
Educational Level	596	3.42
Place of Residence	173	0.99
Province	0	0.00
Age Group	185	1.06
Age	185	1.06

### Types of missingness

Following the fundamental theory of missing data by [1], we present a brief overview of the different missing data mechanisms. Suppose *Y* = {*Y*_*obs*_, *Y*_*mis*_} where *Y*_*obs*_ are the observed values and *Y*_*mis*_ are the unobserved values and let *M* be a missing data indicator matrix of the same dimension as *Y* where the value in row *i* and column *j* is equal to 1 if the value in *Y* is missing and 0 if the value is observed. Data are MCAR if *P*(*M*|*Y*) = *P*(*M*) for all *Y* that is, the fact that the data are missing is not dependent on any values or potential values for any of the variables. That is the probability that a respondent does not report an item value is completely independent of the true underlying values of all the observed and unobserved variables, [7]. Missingness is completely unsystematic and the observed data can be regarded as a random sub-sample of the hypothetically complete data. Thus inference can be carried out with the observed data since they are representative of the complete sample and possibly the target population.

Missing data are MAR if missingness is related to other measured or observed variables in the analysis, but not to the underlying observed values of the incomplete variable, that is the hypothetical values that would have resulted had the data been complete, [5]. Thus MAR implies that *P*(*M*|*Y*) = *P*(*M*|*Y*_*obs*_) for all *Y*. The response mechanism responsible for MCAR and MAR is termed ignorable, [1, 4, 7].

Missing data are MNAR if they are neither MCAR nor MAR, that is if the missing data are not at least MAR. Missing data are MNAR if missingness depends on both the observed and unobserved values of *Y*, that is *P*(*M*|*Y*) = *P*(*M*|*Y*_*obs*_, *Y*_*mis*_) with no further simplification. The MNAR mechanism is also called non-ignorable missing data mechanism.

In the current research the strong MCAR assumption was regarded as not plausible for reasons already stated and instead we adopted the MAR ignorability assumption. Missing data in the HIV variable was perhaps a result of refusal to allow collection of blood samples since HIV issues are still regarded as sensitive in most of sub-Saharan Africa countries. In other variables such as employment status, marital status, contraception, education and literacy levels, missing data were possibly a result of inconsistencies in the responses given for the measured variables.

### Multiple imputations

Formally, following [1], we let *θ* be a population quantity to be estimated, and $\widehat{\theta}=\widehat{\theta}\left({Y}_{obs},{Y}_{mis}\right)$ denotes the statistic that would be used to estimate *θ* if complete data were available and *U* = *U*(*Y*_*obs*_, *Y*_*mis*_) be its variance. In the presence of *Y*_*mis*_ we suppose that we have *m* ≥ 2 independent imputations, *Y*_*mis*_^(1)^, …, *Y*_*mis*_^(*m*)^ the imputed data estimates are calculated as $\widehat{\theta^{(l)}}=\widehat{\theta}\left({Y}_{obs},{Y}_{mis}^{(l)}\right)$ along with their estimated variances *U*^(*l*)^ = *U*(*Y*_*obs*_, *Y*_*mis*_^(*l*)^), *l* = 1, …, *m*. We computed the overall estimate of *θ* as an average given by

1 $$ \overline{\theta}=\frac{1}{m}{\displaystyle \sum_{l=1}^m\widehat{\theta^{(l)}}} $$

In addition, we obtained the standard error of $\overline{\theta}$ as an estimated total variance given by

2 $$ T=\left(1+{m}^{-1}\right)B+\overline{U} $$

where *B* is the between-imputation variance given by

$$ B=\frac{{\displaystyle \sum_{l=1}^m\left(\widehat{\theta^{(l)}}-\overline{\theta}\right)}}{m-1} $$

and *Ū* is the within-imputation variance given by

$$ \overline{U}=\frac{{\displaystyle \sum_{l=1}^m{U}^{(l)}}}{m}\oplus $$

We also provided a confidence interval for the population quantity, *θ* from the combined multiple imputed estimate, $\overline{\theta},$ its standard error and critical value from the Student's *t-*distribution as

$$ CI\left(\theta \right)=\overline{\theta}\pm {t}_{{\overset{\sim }{v}}_{mi}i,1-\raisebox{1ex}{$\alpha $}\!\left/ \!\raisebox{-1ex}{$2$}\right.}\times SE\left(\overline{\theta}\right) $$

where ${\overset{\sim }{v}}_{mi},$ are the degrees of freedom as detailed in [1].

### The analysis model

For both the complete case and the *m* multiple imputed data sets, we considered a survey logistic regression model which is a generalized linear model (GLM), as the analysis model. GLMs, as first introduced by [18] and further expanded by [19] are a unified regression technique for explaining the variations in both normal and non-normal (such as binary) response variables using a set of covariates.

For an illustration of the formulation of the GLMs (and a survey logistic regression model for a binary response variable in particular), suppose *Y*_*i*_ is a binary response variable satisfying the binomial conditions, that is *Y*_*i*_ ~ *Bin*(*n*_*i*_, *π*_*i*_) and let *x*_*i*_ be a vector of predictor variables related to *Y*_*i*_ and can provide additional information for predicting *Y*_*i*_ for *i* = 1, …, *n*. From a GLM perspective, the logistic regression analysis seeks to construct a model that explains the variation in the probabilities *π*_*i*_ using the set of predictors as

3 $$ \pi \left({x}_i\right)={g}^{-1}\left({x}_i^{\hbox{'}}\beta \right) $$

where *β* is a *p-*dimensional set of parameters to be estimated from the data. Thus by a logit transformation

4 $$ \log it\left(\pi \left({x}_i\right)\right)= \log \left(\frac{\pi \left({x}_i\right)}{1-\pi \left({x}_i\right)}\right)={x}_i^{\hbox{'}}\beta $$

Under a complex sampling design, the parameters are estimated via a pseudo-likelihood estimation method as described by [19] rather than the maximum likelihood applicable under the classical GLM. Design-based Wald test statistics are used to test the null hypothesis that *β*_*j*_ = 0 and design-based confidence intervals provide information on the potential magnitude and uncertainty associated with the estimates of each *β*_*j*_ where *j* = 1, …, *p*.

### Statistical computations

We used the multiple imputation method described in Subsection 2.3 above to obtain ‘complete’ data for each of the variables and account for the variability about the missing data. We used the package **mi** in **R** by [20, 21] for the analysis. The package uses a chained equation approach to the imputation, see [22]. The approach allows specification of the conditional distribution of each variable with missing values conditioned on other variables in the data, and the imputation algorithm sequentially iterates through the variables to impute the missing values using the specified models. This is the so called the fully conditional modelling approach [22]. Depending on the variable type with missing values, [21] gave examples of conditional distributions. The multiple imputation procedure was performed using Markov chain Monte Carlo (MCMC) methods making use of an iterative data augmentation technique as explained by [11]. In particular, as described by [21], the basic setup of the multiple imputation procedure in **mi** involves three steps; setup, imputation and analysis. The setup step involves a graphical display of missing data patterns, identifying structural problems in the data and pre-processing as well as specifying conditional models.

In the imputation step, the iterative imputation process was carried out based on the conditional models. The **mi** package handles ‘special’ types of variables with missing values as given by [21]. With reference to the variables in Table 1 above which were used in the imputation model, the package can handle binary variables such as HIV status, place of residence, employment status; ordered categorical variables such as wealth index, literacy level, education level and age group; unordered categorical such as marital status, contraception and religion; and positive continuous such as age. In addition to the main effects we also considered potential interactions that are clinically reasonable and assessed their statistical significance as presented in [23]. Hence we established that there exists an age group by gender interaction effect and it was included in the conditional models. The **mi** package chooses the conditional models automatically according to the variable types identified. In particular, as given in [21], for binary, continuous and ordered categorical, **mi** fits the Bayesian versions of the GLMs (bayesglm). These models are slightly different from the classical GLMs in that they add a Student’s *t-*distribution on the regression coefficients. In the current study we used the default Cauchy distribution as recommended by [24] as given in [21]. Case sampling weights that account for the clustered sample design were included in the conditional models as predictors. Five complete data sets, as suggested in [12] were obtained and analyzed separately using design consistent survey logistic regression models as the analysis models with details as given in Subsection 2.4 utilizing the package **survey** by [25] in **R**. In addition, the **survey** package allows appropriate parameter estimates and their variance estimates, that account for the complex design, to be computed. We combined or pooled the results together using the formulae provided by [1] as explained in Subsection 2.3 above.

## Results

### Prevalence estimation results

We present the design-consistent estimates for HIV prevalence obtained from both a complete case analysis and from the multiple imputed data sets. In the complete case analysis we considered a list-wise deletion of cases with missing values. In the multiple imputation case, the analyses are aimed at accounting for both the complex sampling design and the imputation process. In particular, the variance estimates have to reflect the variability introduced by the imputation process and the variability required to account for the complex sampling design.

Both approaches gave an overall HIV prevalence of approximately 15.7 % However the complete case analysis gave a lower standard error of the estimate of HIV prevalence of 0.32 % as compared to 0.39 % for the multiple imputations. For the overall prevalence in particular, the larger standard error for the multiple imputation approach correctly incorporates the between and within imputation variances, as we can never know the true value of the missing data as explained by [2].

Results of the crude subgroup estimates of HIV prevalence are given in Table 2. The results in the table show that the estimates obtained from both the complete case and the imputation are not identical. This is possibly because of the additional 2 943 cases that the multiple imputations have allowed to enter the analysis. However the differences are not statistically significant as the 95 % confidence intervals from the two approaches overlap except for the estimate for the single/never married respondents under the variable gender. The estimated standard errors of the estimates for the multiple imputation case are generally less than those for the complete case analysis. This possibly reflects the effect of the recovered additional information, by multiple imputations, from the incomplete cases that were ignored under the list-wise deletion. The confidence intervals for the multiple imputations are generally tighter than those from the complete case analysis. This reflects the effects of the extra precision that multiple imputations introduce in the estimation process. The results in Table 2 generally correspond to the results published in the 2010–11 Zimbabwe Demographic and Health Surveys report.

**Table 2:** Overall and subgroup estimates and their standard errors of HIV prevalence for (a) complete case analysis and (b) multiple imputation

Variable	(a) Complete case analysis			(b) Multiple imputations		
	Estimate	S. E.	95 % CI	Estimate	S. E.	95 % CI
Overall	0.157	0.0032	(0.147, 0.160)	0.157	0.0039	(0.150, 0.164)
Gender						
Male	0.128	0.0045	(0.118, 0.137)	0.131	0.0042	(0.123, 0.139)
Female	0.177	0.0045	(0.166, 0.187)	0.178	0.0045	(0.169, 0.188)
Age Group						
15 - 19	0.040	0.0036	(0.032, 0.047)	0.041	0.0032	(0.035, 0.048)
20 – 24	0.079	0.0053	(0.068, 0.089)	0.085	0.0053	(0.076, 0.095)
25 – 29	0.158	0.0078	(0.142, 0.173)	0.160	0.0073	(0.146, 0.174)
30 – 34	0.232	0.0100	(0.213, 0.252)	0.233	0.0100	(0.214, 0.252)
35 – 39	0.269	0.0120	(0.245, 0.292)	0.272	0.0129	(0.251, 0.294)
40 – 44	0.255	0.0139	(0.228, 0.283)	0.249	0.0125	(0.227, 0.272)
45 – 49	0.258	0.0153	(0.227, 0.288)	0.265	0.0151	(0.263, 0.294)
50 - 54	0.187	0.0224	(0.143, 0.231)	0.191	0.0201	(0.154, 0.229)
Marital Status						
Single	0.056	0.0034	(0.049, 0.063)	0.083	0.0031	(0.076, 0.091)
Married	0.167	0.0044	(0.159, 0.177)	0.169	0.0039	(0.159, 0.179)
Divorced	0.288	0.0162	(0.256, 0.319)	0.276	0.0120	(0.259, 0.323)
Widowed	0.544	0.0221	(0.500, 0.587)	0.551	0.0203	(0.510, 0.587)
Wealth Index						
Poorest	0.151	0.0073	(0.143, 0.172)	0.159	0.0062	(0.142, 0.176)
Poorer	0.158	0.0072	(0.132, 0.161)	0.148	0.0052	(0.134, 0.162)
Middle	0.146	0.0076	(0.149, 0.179)	0.138	0.0074	(0.150, 0.187)
Richer	0.163	0.0069	(0.146, 0.174)	0.170	0.0058	(0.155, 0.184)
Richest	0.142	0.0066	(0.126, 0.152)	0.142	0.0065	(0.129, 0.154)
Literacy						
Non-lit	0.139	0.0124	(0.115, 0.162)	0.149	0.0158	(0.122, 0.176)
Partially	0.198	0.0137	(0.172, 0.223)	0.194	0.0123	(0.171, 0.217)
Literate	0.151	0.0030	(0.144, 0.157)	0.155	0.0041	(0.147, 0.162)
Employment						
Yes	0.135	0.0041	(0.128, 0.143)	0.139	0.0041	(0.130, 0.147)
No	0.173	0.0050	(0.163, 0.183)	0.177	0.0049	(0.166, 0.187)
Place of Res						
Rural	0.147	0.0038	(0.139, 0.154)	0.148	0.0035	(0.138, 0.157)
Urban	0.168	0.0059	(0.157, 0.180)	0.172	0.0054	(0.160, 0.184)

### Logistic regression results

We present the results of a survey logistic regression model (as the analysis model) with estimates and their standard errors pooled from the multiply imputed data sets using the formulae provided by [1] as well as results from the complete case analysis. Specifically, we fitted survey logistic regression model to explain or model the variation in HIV prevalence as a function of demographic and socio-economic variables while accounting for the complex sampling design. We established that although HIV prevalence generally increases with age for both males and females, the rates of the increases are not the same, hence the inclusion of the age by gender interaction effect (effect modification). The results are displayed in Table 3 as adjusted odds ratios for the estimates of the logistic regression models obtained under each of the two approaches. For the interpretation of the odds ratios, the reference level approach was adopted. The odds ratios for each covariate were adjusted for the other covariates in the models. In particular the odds ratios show the multiplicative effect of each given level, as the likelihood of being HIV positive, of a covariate relative to a reference level controlling for the effect of the other covariates in the model.

**Table 3:** Adjusted odds ratios for the survey logistic regression models under (a) complete case analysis and (b) multiple imputations analysis

Paramter	(a) Complete case analysis		(b) Multiple imputations analysis	
	OR	95 % CI	OR	95 % CI
Intercept	0.030	(0.021, 0.041)	0.037	(0.025, 0.055)
Gender				
Female	Ref.		Ref.	
Male	0.924	(0.631, 1.354)	0.812	(0.561, 1.175)
Age Group				
15 – 19	Ref.		Ref.	
20 – 24	2.314	(1.689, 3.171)	2.659	(2.000, 3.534)
25 – 29	4.478	(3.293, 6.089)	5.380	(4.029, 7.183)
30 – 34	6.286	(4.730, 8.857)	7.957	(5.948, 10.643)
35 – 39	6.472	(4.550, 8.684)	7.936	(5.797, 10.865)
40 – 44	3.981	(2.778, 5.705)	5.007	(3.511, 7.140)
45 – 49	3.171	(2.191, 4.589)	4.453	(3.127, 6.340)
50 - 54	4.325	(2.746, 6.812)	4.468	(1.050, 19.019)
Marital Status				
Single	Ref.		Ref.	
Married	1.182	(0.973, 1.437)	0.842	(0.726, 0.976)
Divorced	2.535	(1.990, 3.230)	1.658	(1.238, 2.220)
Widowed	6.605	(5.017, 8.695)	4.464	(3.581, 5.564)
Literacy				
Non literate	Ref.		Ref.	
Partially	1.662	(1.258, 2.194)	1.503	(1.094, 2.064)
Literate	1.280	(1.022, 1.602)	1.194	(0.905, 1.576)
Place of Residence				
Rural	Ref.		Ref.	
Urban	1.251	(1.117, 1.401)	1.224	(1.084, 1.382)
Age Group*Gender				
15 – 19:Male	Ref.		Ref.	
20 – 24:Male	0.447	(0.268, 0.745)	0.566	(0.346, 0.925)
25 – 29:Male	0.583	(0.370, 0.919)	0.637	(0.417, 0.972)
30 – 34:Male	0.653	(0.416, 1.026)	0.732	(0.449, 1.194)
35 – 39:Male	1.044	(0.662, 1.648)	1.129	(0.751, 1.696)
40 – 44:Male	1.818	(1.109, 2.979)	1.808	(1.069, 3.058)
45 – 49:Male	2.734	(1.636, 4.571)	2.498	(1.578, 3.955)

## Discussion

The results for the two approaches presented in Tables 2 and 3 are not identical although they are generally consistent pertaining to the statistical interpretation of the estimates. In particular, the crude estimates of HIV prevalence presented in Table 2 show no statistical significant differences between the two approaches. This is particularly so because the respective 95 % confidence intervals for the estimates overlap. The results consistently show that the risk of HIV is lower among males $\Big(\widehat{p}=12.8\%,$ 95 % *CI* = 11.8 − 13.7 % for the complete case analysis and $\widehat{p}=13.1\%,$*CI* = 12.3 − 13.9 % for the multiple imputations) than among females $\Big(\widehat{p}=17.7\%,$ 95 % *CI* = 16.6 − 18.7 % for the complete case analysis and $\widehat{p}=17.8\%,$ 95 % *CI* = 16.9 − 18.8 %). The differences are possibly due to the disparities in susceptibility to HIV between females and males especially in light of HIV infection through unprotected heterosexual intercourse. It has been reported that the risk of transmitting HIV from men to women is much higher than from women to men because women are exposed to considerable amounts of seminal fluids during vaginal sexual intercourse [26, 27]. Both approaches show a general increase in HIV prevalence with age peaking at the same age group 35–39 HIV prevalence is least among the single or never married for both approaches although the difference in the prevalence between the two is statistically significant as the 95 % confidence intervals do not overlap. In particular, the prevalence is significantly lower $\Big(\widehat{p}=5.6\%,$ 95 % *CI* = 4.9 − 6.3 %) under the complete case analysis than under the multiple imputation $\Big(\widehat{p}=8.3\%,$ 95 % *CI* = 7.6 − 9.1 %). The widowed have the highest HIV prevalence for both approaches and there is no statistical significant difference in the prevalence between the two approaches as the 95 % confidence intervals overlap. The interpretation of the results is the same for the other risk factors indicated in Table 2.

With reference to Table 3 both approaches show that the risk of HIV is less among the males (*OR* = 0.924, 95 % *CI* = 0.631 − 1.354 under the complete case analysis and *OR* = 0.812, 95 % *CI* = 0.516 − 1.175 under the multiple imputation) compared to the females controlling for the other covariates in the model. However both approaches show that the difference in the risk among males and females is not statistically significant as the both confidence intervals include 1. The results show that the risk of HIV increases with age for both approaches, however the multiple imputation results show higher risk at every age group. Relative to the single/never married, the married are slightly more likely to be HIV positive (*OR* = 1.182, 95 % *CI* = 0.973 − 1.437) under the complete case analysis, whereas the married are slightly less likely (*OR* = 0.842, 95 % *CI* = 0.726 − 0.976) under the multiple imputations controlling for the other covariates in the model. The divorced are twice more likely (*OR* = 2.575, 95 % *CI* = 1.990 − 3.230) under the complete case, whereas they are less than twice more likely (*OR* = 1.658, 95 % *CI* = 1.238 − 2.220) to be HIV positive relative to the single/never married controlling for the other covariates in the model. The interpretations are the same for literacy levels and the place of residence.

The married level of marital status variable ceased to be non-significant under complete case analysis to being significant under multiple imputations whereas the literate level of the literacy variable ceased to be significant under the complete case analysis to being non- significant under the multiple imputation analysis. The age by gender interaction effect shows that the risk of HIV is significantly higher, as evidenced by 95 % confidence intervals that are not overlapping, in females than in males among the young age groups. However the risk is higher among males in age group 40–44 year olds and significantly higher among the 45–49 year olds in males than in females. These findings agree well with a general perception in most sub-Saharan African countries that younger women engage in sexual activities with older men a key driver of HIV infection in sub-Saharan Africa.

## Potential strength and limitations of the study

The research draws its strength from the use of the multiple imputation technique to impute missing data in HIV research utilizing the powerful and advanced computational tools that are now available in statistical software such as **R**. Also noting that missing data are inevitable, pervasive and have severe consequences if not properly handled, use of sound statistical methods and computing resources to estimate disease measures of interest and appropriate measures of variability (that account for both the sampling mechanism and the imputation process) can enhance the validity of the statistical interpretations and inferences.

However a potential drawback of the current research comes from the use of secondary data which often leaves the data analyst with limited control over the data collection process. In addition, and particularly for the current research, a major drawback of using secondary is the limited knowledge about the reasons for the missing values. However this is not to downplay the importance of DHSs which are carefully designed, by a team of highly trained statisticians with excellent expertise in survey methodology, to collect population level information which is very important for public health policies. The package **mi**, although very powerful and flexible, comes with its own limitations that it cannot allow users to alter the prior distributions for the conditional imputation models used under the Bayesian paradigm. Therefore further methodological and software developments research is necessary in order to make the approach even more flexible. Further work on the problem as a future extension is possible with inclusion of methods that allow for MNAR assumption by means of sensitivity analysis.

## Conclusion

Analysis of survey data that are characterized by missing data often take a complete case analysis approach where cases with missing values are excluded in the analysis. This often introduces bias in the estimates because of potential loss of information that occurs with the deletion of the cases with missing values. Alternatively, ad hoc approaches based on substituting the missing values with plausible ones such as the last value carried forward, the mean and the regression predictions (single imputations) can be used. However, these approaches may result in potential loss of the distributional relationships amongst variables and it is not possible to provide measures of uncertainty introduced by the imputation process. Hence we utilized the multiple imputation procedure to ‘fill in’ missing values and obtain unbiased estimates of HIV prevalence in Zimbabwe using the 2010–11 DHS data while at the same time accounting for the uncertainty about the missing data themselves. Crude design-consistent national and subgroup estimates of HIV prevalence were estimated under both the complete case analysis and the multiple imputation analysis. Survey logistic regression models were also fitted and the results showed considerable variation in the estimates obtained under the two approaches. The results of both the crude estimates and the survey logistic regression model show substantial differences in the estimates and the widths of the confidence intervals between the two approaches.

## Abbreviations

AIDS: Acquired immune deficiency syndrome
CI: Confidence interval
CDC: Center for disease control
DBS: Dried blood spot
EA: Enumeration area
ELISA: Enzyme-linked immunosorbent assays
GLM: Generalized linear model
HIV: Human immunodeficiency virus
MAR: Missing at random
MCAR: Missing completely at random
MCMC: Markov chain Monte Carlo
MNAR: Missing not at random
NMRL: National microbiology reference laboratory
SE: Standard error
STROBE: Strengthening the reporting of observational studies in epidemiology
VCT: Voluntary counseling and testing
ZDHS: Zimbabwe demographic and health surveys
